# Absence of IFNγ promotes hippocampal plasticity and enhances cognitive performance

**DOI:** 10.1038/tp.2015.194

**Published:** 2016-01-05

**Authors:** S Monteiro, F M Ferreira, V Pinto, S Roque, M Morais, D de Sá-Calçada, C Mota, M Correia-Neves, J J Cerqueira

**Affiliations:** 1 Life and Health Sciences Research Institute, School of Health Sciences, University of Minho, Campus de Gualtar, Braga, Portugal; 2 Life and Health Sciences Research Institute, 3B's - PT Government Associate Laboratory, Braga/Guimarães, Portugal

## Abstract

Cognitive functioning can be differentially modulated by components of the immune system. Interferon-γ (IFNγ) is a pro-inflammatory cytokine whose production is altered in many conditions displaying some degree of cognitive deficits, although its role in cognitive functioning is still unclear. Here we show that the absence of IFNγ selectively enhances cognitive behaviours in tasks in which the hippocampus is implicated. Moreover, the absence of IFNγ leads to volumetric and cell density changes that are restricted to the dorsal part of the hippocampus. In the dorsal hippocampus, the absence of this pro-inflammatory cytokine leads to an increase in the numbers of newly born neurons in the subgranular zone of the dentate gyrus (DG), an adult neurogenic niche known to support learning and memory, and to an enlargement of the dendritic arborization of DG granule and cornu ammonis *(*CA)1 pyramidal neurons. Moreover, it also modestly impacts synaptic plasticity, by decreasing the paired-pulse facilitation in the Schaffer collateral to CA1 pyramidal cell synapses. Taken together, our results provide evidence that IFNγ is a negative regulator of hippocampal functioning, as its absence positively impacts on dorsal hippocampus structure, cell density, neuronal morphology and synaptic plasticity. Importantly, these neuroplastic changes are associated with improved performance in learning and memory tasks. Therefore, blockage of the IFNγ signalling may present as promising therapeutic targets for the treatment of inflammation-associated cognitive dysfunction.

## Introduction

The modulation of cognitive functioning by immune mediators is receiving increasing attention, as a common inflammatory component is recognized in many neurodevelopmental and neuropsychiatric disorders. The association of cytokines with cognitive functioning is underpinned by studies showing the differential action of these molecules on learning and memory,^1, 2, 3, 4^ synaptic plasticity^5, 6, 7, 8^ and neurogenesis.^1, 2, 3, 4, 6, 9, 10, 11, 12, 13, 14^ One of the most relevant cytokines in these interactions is interferon-γ (IFNγ), a pro-inflammatory mediator produced mainly by T, natural killer and NT cells, whose levels are altered with chronic stress exposure,^15^ ageing^16, 17^ and in several neuropsychiatric^15, 17^ and neurodevelopmental disorders,^18^ all of which present, to some extent, with cognitive impairment. Several studies have demonstrated that IFNγ is able to impact brain function by affecting systems that are based on cognitive behaviours. For instance, different studies have highlighted the important role of IFNγ on the regulation of neural precursors cells.^9, 11, 12, 13^ Indeed, it was shown that neural precursor cells express the IFNγ receptor, and that IFNγ reduces the viability of neural precursor cells and increases cell death through caspase-3 expression.^19^ Furthermore, it has been shown that mice lacking IFNγ display increased neurogenesis in the subventricular zone,^9^ an adult neurogenic niche. Controversially, there are also some studies showing that IFNγ can increase proliferation of neural precursor cells,^12^ or enhance neurogenesis in an Alzheimer's disease mouse model.^20^ In fact, it is known that IFNγ can have opposing effects on inhibiting or promoting proliferation depending on the molecular pathways activated.^21^ For instance, it has been shown that the increase on neural precursor cells' proliferation mediated through the sonic hedgehog pathway is dysfunctional in these mice leading to the differentiation of aberrant cells.^11^

IFNγ was also associated with disturbances of the serotonergic signalling, as this cytokine activates the rate-limiting enzyme for tryptophan—indoleamine-pyrrole 2,3-dioxygenase a serotonin precursor. In fact, disturbances of tryptophan metabolism caused by IFNγ have been discussed in the context of depression,^22^ ageing and ageing-related neuropsychiatric disorders.^17^ Moreover, kynurenine metabolites which result from indoleamine-pyrrole 2,3-dioxygenase activity, such as 3-hydroxy-kynurenine and quinolinic acid, are known to be toxic to the brain.^22, 23^

Another line of research has consistently shown the importance of T lymphocytes in supporting cognition.^24, 25, 26, 27, 28, 29^ For instance, training in a cognitive task was shown to induce the accumulation of interleukin-4 producing T cells in the meninges, and conversely, mice lacking this cytokine exhibit cognitive deficits together with a skewed pro-inflammatory meningeal phenotype.^2^ These are very interesting findings considering that one of the known biological functions of interleukin-4 is the suppression of Th1 differentiation—cells for which IFNγ is considered the main effector cytokine.

Despite the above-mentioned studies and others showing that IFNγ can impact different brain functions, the role of IFNγ as a modulator of cognitive performance in the healthy brain is far from being understood. Therefore, we set out to (1) understand whether IFNγ is indeed a regulator of cognitive performance; (2) study the impact of IFNγ on structural and electrophysiological correlates of cognitive functioning in the hippocampus.

Herein we show that the absence of endogenous IFNγ leads to increased neurogenesis and dendritic length in the dorsal hippocampus that correlates with enhanced performance in learning and memory tasks; in addition, our results provide evidence of a brain regional selectivity for endogenous IFNγ action with the dorsal, but not the ventral, part of the hippocampus exhibiting the most significant alterations caused by the absence of this cytokine.

## Materials and methods

### Animals

Three-to-five-month-old female IFNγ knockout (KO) mice on C57BL/6 background^30^ and littermate homozygous wild-type (WT) were housed (five per cage) under standard laboratory conditions (12 h light/12 h night cycles (08 h/20 h)), 22–24 °C, relative humidity of 55% and with *ad libitum* access to water and food. Cages were enriched with paper rolls and soft paper for nesting. All the procedures were carried out in accordance to EU directive 2010/63/EU and Portuguese national authority for animal experimentation, Direção Geral de Veterinária (ID:DGV9457), guidelines on animal care and experimentation. Littermate WT were used as controls in all the experiments to minimize genetic variability^31^ and were obtained from an initial crossing of IFNγ KO mice with C57BL/6 mice (Charles River Laboratories, Barcelona, Spain) and by crossing the heterozygous progeny between them. Mice were killed at 5 months of age. To exclude the possible impact of hormonal variability on the results, female mice were only killed whenever they were at proestrus phase.

### Behavioural assessment

Behavioural experiments were performed during the mice-active period (between 2000 and 0800 h). Before behavioural testing, mice were gently handled by the same experimenter for 2 weeks every other day. Before each behavioural assessment, mice were transported to the testing room and left for habituation to room conditions for 1 h. All behavioural data analysis was performed with the experimenter blinded to the genotype.

#### Morris water maze

To assess spatial reference memory, 3-month-old mice were tested in a white circular pool (170 cm diameter) filled with water (24–25 °C) placed in a dimly lit room. Spatial cues were placed in the walls around the pool (square, stripes, triangle and a cross). The pool was divided into four imaginary quadrants and a hidden transparent platform was placed in one of the quadrants. Data were collected by a fixed camera placed in the ceiling and connected to a video-tracking system (Viewpoint, Champagne-au-Mont-d'or, France).

The mice had to learn the position of a hidden platform over a period of 4 days. Each day, the mice were placed facing the wall of the pool at different quadrants (north, west, south and east), in a pseudorandom order that varied from day to day, as a starting point for each trial. Each trial was completed whenever the mouse reached the platform or when 120 s elapsed. Latency to reach the platform (escape latency) was recorded for each trial during the 4 days. On the fifth day, the platform was removed and a single trial of 60 s was performed (probe trial). The percentage of time that each mouse swam in each quadrant was recorded to confirm the acquisition of platform location through reference memory. Behavioural flexibility was assessed in the reversal task that took place on the fifth day immediately after the probe trial; briefly, the platform was positioned in a new (opposite) quadrant and the animals had three consecutive 120-s trials to find it in the new position. Latency to reach the platform (escape latency) in the new location was recorded for each trial.

#### Novel object recognition

Recognition memory was evaluated by submitting animals to the novel object recognition test. Briefly, 3-month-old mice were habituated to a black box for 1 h in three consecutive days. On the fourth day, two similar objects were symmetrically placed on one side of the box and the animals were allowed to freely explore both the objects for 10 min. The mice were then immediately returned to their home cages and the box and objects were cleaned with 10% ethanol solution. The test session was carried out 1 h later. One of the objects was replaced by a novel one (similar size but different colour and shape); each mouse was then reintroduced into its original test box where it was allowed to freely explore both the objects for 5 min. The exploration time in each object was recorded. The animals were considered to be exploring whenever the nose was facing the object. Recognition memory was assessed by the discrimination ratio, that is, time exploring the novel object minus time exploring the old object over the total exploration time.

#### Elevated-plus maze

Three-month-old mice were tested for anxiety-like behaviour using the elevated-plus maze test. Briefly, this test consists on placing each mouse in the hub of a plus-like apparatus elevated 72.4 cm from the floor, with two opposing open arms (50.8 cm × 10.2 cm) and two opposing closed arms (50.8 cm × 10.2 cm × 40.6 cm; ENV560; MedAssociates, St. Albans, VT, USA) and letting the animal freely explore it for 5 min. Time in the open arms and in the closed arms was used as a behavioural parameter of anxiety-like behaviour.

#### Open field

Three-month old mice were tested for locomotor activity and anxiety-like behaviour using the open field. Each mouse was left in the centre of a square arena (43.2 cm × 43.2 cm), which the mouse was free to explore for 5 min. This arena was equipped with infrared beams for activity detection (MedAssociates). Data were collected using the activity monitor software (MedAssociates). Distance travelled was used as a measure of locomotor activity. The ratio between time spent in the centre (10.8 cm × 10.8 cm) and periphery of the arena was used as a measure of general anxiety.

### Determination of oestrous cycle stage

Vaginal smears were performed by inserting a drop of sterile 0.9% saline solution in the vagina with the help of a 1 ml syringe, collecting the cell suspension by inserting a small plastic inoculation loop and performing a smear into a glass slide. Smears were air-dried, fixed in alcohol 96% for 5 min and stained using the Papanicolaou protocol. Briefly, smears were hydrated in tap water, stained with Harris haematoxylin for 1 min, rinsed in running tap water for 2 min, regressively stained by a single dip in a alcohol–acid solution, rinsed in tap water for 2 min, dehydrated in alcohol 96% for 1 min, stained with orange G for 1 min, washed in alcohol 96% for 1 min, stained with Eosine Azure 50 for 1 min, dehydrated in a decreasing series of alcohol concentration and cleared with xylene. Slides were analysed under a light microscope and the proportion of cornified epithelial cells, nucleated epithelial cells and leukocytes was used for the determination of the oestrous cycle phases.^32^

### Hippocampal formation volumes

Five-month-old mice were anaesthetized with 20% sodium pentobarbital (Eutasil, 100 mg kg^−1^; Ceva, Algés, Portugal) and transcardially perfused with 0.9% saline solution and 4% paraformaldehyde (PFA). After being removed, brains were further fixed in 4% PFA solution with constant agitation for 24 h and kept in 4% PFA until tissue processing (~4 weeks). Brains were processed for stereology according to the method described previously.^33^ Briefly, brains were embedded in glycolmethacrylate (Tecnovit, 7100; Heraeus Kulzer, Werheim, Germany) and 30 μm-thick sections were obtained using a microtome. Every other section was collected into non-coated glass slide, stained with Giemsa and coverslipped using entellan (Entellan New, Merck, Darmstadt, Germany). To minimize bias, each brain was coded to keep the experimenter blind to the genotype. The hippocampal formation was analysed according to its main anatomical divisions: dentate gyrus (DG), cornu ammonis (CA) 3 and CA1 (strata oriens, pyramidale and radiatum). The hippocampal formation analysis was further divided into dorsal and ventral parts. Volumes of the different hippocampal subregions were determined using the Cavalieri's principle.^34^ Briefly, every fourth section was used and it's cross-sectional area was estimated by point counting. For this, we randomly superimposed onto each area a test point grid in which the interpoint distance, at a tissue level, was 150 μm for the DG and 200 μm for CA3 and CA1 regions. The volume of the region of interest was calculated from the number of points that felt within its boundaries and the distance between the systematically sampled sections. Average neuronal cell density numbers was estimated using the optical fractionator method.^35^ Briefly, as for volumes, every fourth section was selected and the boundaries of every hippocampal subregion were defined as stated above. A grid of virtual three-dimensional boxes for granule cell layer of the DG (20 × 20 × 20) and for pyramidal cell layer of CA1 and CA3 regions (40 × 40 × 40) was superimposed onto each section and neurons were counted whenever their nucleus (identified by size, shape and a prominent nucleoli) came into focus within the counting box.

Volume and neuronal number estimations were performed using Newcast software (Visiopharm, Horsholm, Denmark) and a camera attached to a motorized microscope (BX51, Olympus, Tokyo, Japan).

### Immunofluorescence

Five-month-old mice were deeply anaesthetized with 20% sodium pentobarbital and were transcardially perfused with cold 4% PFA. The brains were removed and post-fixed in 4% PFA under constant agitation for 24 h, immersed in 30% sucrose solution, preserved with optimal cutting temperature compound and snap-frozen. Serial coronal sections (20 μm) were cut in a cryostat (Leica CM1900, Leica Biosystems, Nussloch, Germany) and collected on slides for immunofluorescence. Sections were stained for proliferating cells with anti-Ki-67 polyclonal antibodies (1:300, AB9260, Millipore, Darmstadt, Germany) followed by staining for neuroblasts with polysialic acid-NCAM antibodies (1:300, clone 2-2B, MAB5324, Millipore). Finally, for nuclear staining, all the sections were incubated with 4',6- diamidino-2-phenylindole (1:1000, Sigma-Aldrich, St. Louis, MO, USA). For each animal, Ki-67-positive cells double stained with polysialic acid-NCAM antibodies within the subgranular zone (SGZ) of the dorsal DG were counted using confocal microscopy (Olympus FluoView FV1000, Hamburg, Germany). For assessing neurogenesis (differentiation), we counted the number of neuroblasts (double-positive cells) divided by the total number of proliferating cells (Ki-67^+^). We performed this analysis in the dorsal hippocampus SGZ, including at least four sections per animal, from the dorsal hippocampus region (bregma −1.58 and −2.06). To minimize bias, each slide was coded to keep the experimenter blind to the genotype.

### Hippocampal neurons morphology

To assess the three-dimensional (3D) dendritic morphology of hippocampal neurons, we used the Golgi–Cox method. Briefly, 5-month-old mice were anaesthetized with 20% sodium pentobarbital and transcardially perfused with 0.9% saline solution. The brains were removed and immersed in Golgi–Cox solution for 21 days and then transferred to a 30% sucrose solution and cut on a vibratome. Coronal sections (200 μm thick) were collected in 6% sucrose and blotted dry onto gelatin-coated microscope slides. They were subsequently alkalinized in 18.7% ammonia, developed in Dektol (Kodak, Rochester, NY, USA), fixed in Kodak Rapid Fix, dehydrated, xylene cleared, mounted and coverslipped with entellan. All incubation steps were performed in a dark room. To minimize bias, each brain was coded to keep the experimenter blind to the genotype. The 3D reconstruction of Golgi-impregnated neurons from the DG and CA1 were evaluated. Five to eight neurons were randomly selected for 3D reconstruction having the following criteria in consideration: (1) full Golgi-impregnation along the dendritic tree; (2) complete dendrites without truncated branches; and (3) relative isolation from neighbouring impregnated neurons, astrocytes or blood vessels to avoid interference with the analysis. Slides containing the region of interest were randomly searched and the first five to eight neurons fulfilling the criteria (maximum of three neurons per section) were selected. For each selected neuron, all branches of the dendritic tree were reconstructed at × 600 (immersion oil) magnification using a motorized microscope (BX51 Olympus), with a camera attached (DXC390; Sony, Tokyo, Japan) and Neurolucida software (MicroBright Field, Williston, VT, USA). A 3D analysis of the reconstructed neurons was performed using NeuroExplorer software (MicroBright Field). Dendritic morphology was examined by the total dendritic length and arrangement of dendritic material using a 3D version of Sholl analysis^36^ of intersections. The number of dendritic intersections with concentric spheres positioned at radial intervals of 10 μm from the soma was registered.

### Electrophysiological recordings

#### Slice preparation

Three-month old mice were killed by decapitation after deep anaesthesia with sodium pentobarbital (30 mg kg^−1^). The brains were quickly removed and placed in ice-cold sucrose-based artificial cerebrospinal fluid (ACSF) containing the following solution: 2.5 mm KCl, 7 mm MgCl_2_, 1.25 mm NaH_2_PO_4_, 110 mm sucrose, 25 mm NaHCO_3_, 7 mm glucose, bubbled with carbogen gas (95% O_2_, 5% CO_2_). After a hemisection of the brain along the sagittal plane, the dorsal hippocampus of the right hemisphere was partially dissected and glued vertically with the dorsal-most part facing up. Horizontal slices (300 μm) were prepared in sucrose-based ACSF using a tissue slicer (Leica VT 1200 s; Leica Biosystems) and incubated for 20 min at 30 °C in standard ACSF containing: 124 mm NaCl, 4.4 mm KCl, 1 mm MgSO_4_, 2 mm CaCl_2_, 1 mm NaHCO_3_, 10 mm glucose, bubbled with carbogen gas. The slices were stored in ACSF at room temperature for at least 30 min before recording, after which they were transferred to a submerged chamber, maintained at 31 °C and continuously perfused with ACSF at a rate of 5 ml min^−1^. Two slices per animal were used for electrophysiological recordings.

#### Electrophysiological recording

Extracellular field recordings were made with a Multiclamp 700B amplifier (Molecular Devices, Sunnyvale, CA, USA) in bridge mode and digitized with the Digidata 1440 digitizer (Molecular Devices) using pCLAMP10 software (Molecular Devices). Signals were low-pass filtered at an effective corner frequency of 3 kHz and sampled at 10 kHz. For recording, borosilicate glass recording pipettes (3–5 m) were pulled using a micropipette puller (P-97, Sutter Instruments, Novato, CA, USA) and filled with saline solution (0.75 m NaCl). For stimulation of the Schaffer collaterals, a stimulus-isolating unit (STG4002, Multichannel Systems, Reutlingen, Germany) and a custom-made bipolar tungsten electrode (Science Product, Hofheim, Germany) were used. Both recording and stimulating electrodes were placed in the middle of CA1 stratum radiatum. The frequency of baseline stimulation was of 0.03 Hz and for input–output relation monitoring, a series of increasing stimulus intensities were applied (0.5–8.0 V). Stimulus strength was then adjusted to have ~40% of the maximum slope of the local field excitatory postsynaptic potential (EPSP). The paired-pulse ratio was assessed before long-term potentiation (LTP) induction by giving two close stimuli of varying inter-pulse intervals (25, 50, 100 and 300 ms).^37^ The ratio was calculated by dividing the slope of field EPSP2 by the slope of EPSP1. For LTP induction, field EPSP slopes were monitored for a period of at least 20 min. If synaptic transmission was stable, three bursts of 1 s duration, separated by 15 s, and with a burst frequency of 100 Hz, were delivered and followed by 80 min of baseline recording. All points of each individual curve were normalized to the average value of baseline. All stored traces were averages of four consecutive recordings. Final slopes were calculated offline using the LTP software.^38^ To minimize bias, the experimenter who performed all the electrophysiology experiments was blinded to the genotype.

### Statistical analysis

Adequate sample size was determined *a priori* using G-Power software v3.1.9.2, based on results of a previous pilot experiment suggesting a *η*^2^_p_ of 0.15 for the effect of genotype on the Morris water maze test and assuming a 90% power and 5% probability of type I errors. All the values were calculated as means+s.e.m. Kolmogorov–Smirnov normality test was used to assess whether data presents a normal distribution. Equality of variances was tested with an F test. Data from the reference memory, reverse task, sholl analysis and electrophysiology were analysed using analysis of variance repeated-measures and differences between groups compared with the *post hoc* Bonferroni test. To quantify the strength of the differences, partial eta-square (*η*^2^_p_) was calculated as a measure of effect size (0.02 was considered a small-, 0.13 a medium- and 0.26 a large-effect size). All the other data were analysed using two-tailed Student's *t*-test and Cohen *d* was calculated as a measure of effect size (0.2 was considered a small-effect size, 0.5 a medium-effect size and 0.8 a large-effect size). Differences were considered significant if *P*<0.05. Statistical analyses were performed with Graphpad Prism version 5.0b (La Jolla, San Diego, CA, USA).

## Results

### Absence of IFNγ leads to an enhanced performance in cognitive tasks that engage the hippocampus

**Figure 1:**
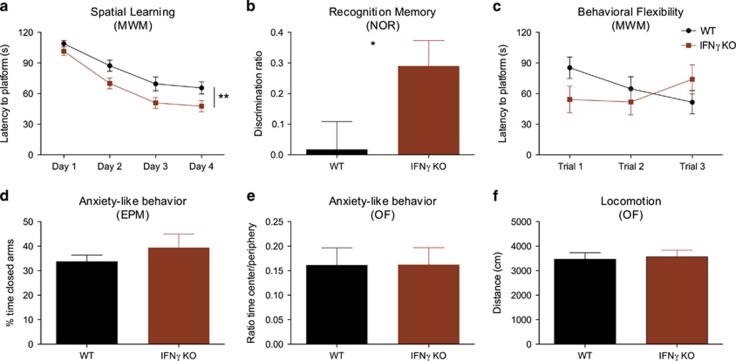
Spatial learning and recognition memory are enhanced in IFNγ KO mice. (**a**) Hippocampal-dependent spatial learning was tested in the MWM (IFNγ KO *n*=23 and WT=26). (**b**) Hippocampal-dependent non-spatial recognition memory was assessed in the NOR task (IFNγ KO *n*=12 and WT=12). (**c**) Prefrontal cortex-dependent behavioural flexibility was assessed in the reversal task (IFNγ KO *n*=23 and WT=26). Anxiety-like behaviour was assessed using the (**d**) EPM (IFNγ KO *n*=7 and WT=10) and (**e**) OF (IFNγ KO *n*=7 and WT=10). (**f**) Motor activity was assessed in the OF (IFNγ KO *n*=7 and WT=10). Error bars denote s.e.m.; *P<0.05; **P<0.01. EPM, elevated-plus maze; IFNγ, interferon-γ KO, knockout; MWM, Morris water maze; NOR, novel object recognition; OF, open field; WT, wild type.

We tested cognitive behaviour of IFNγ KO mice and WT in the Morris water maze using two different paradigms: the spatial reference memory task (Figure 1a) to test learning that relies on hippocampal functioning (during four consecutive days), and the reversal task (on the fifth day) for testing behavioural flexibility (Figure 1c), a prefrontal cortex function.^39^ Both IFNγ KO and WT mice were able to successfully learn the spatial reference memory task (time: F_(3,141)_=43.1; *P*<0.0001; *η*^2^_p_=0.48), but animals lacking IFNγ had a significantly better performance (genotype: F_(1,47)=_10.47; *P*=0.002; *η*^2^_p_=0.13; *d*_(day4)_=0.63; interaction: F_(3,141)_=0.61; *P*=0.60; Figure 1a). Interestingly, this effect seemed to be specific for hippocampal-dependent tasks as in the reversal task there were no differences between groups (Figure 1c). To further clarify this specificity, we used the novel object recognition test to evaluate non-spatial recognition memory, a different dimension of hippocampal functioning. In accordance with our previous results, IFNγ KO mice also exhibited a better performance than WT in this test (*t*_(22)_=2.21; *P*=0.03; *d*=0.90; Figure 1b).

To clarify whether the impact of IFNγ absence is specific for cognitive-related tasks (more related with dorsal hippocampal functioning) or it also affects non-cognitive dimensions such as anxiety-like behaviour (to which the ventral hippocampus is more relevant), we tested the animals in the elevated-plus maze and the open field (Figures 1d and e) and observed no significant differences between the experimental groups. There were also no differences between groups in total distance travelled therefore validating behavioural tests that are dependent on an intact locomotor function (Figure 1f).

### Absence of IFNγ induces structural changes in the dorsal hippocampus

**Figure 2:**
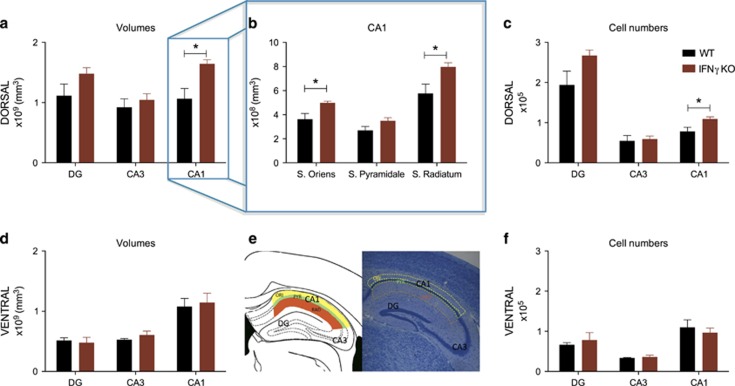
The structure of the dorsal but not the ventral hippocampus is altered in IFNγ KO mice. Stereological estimations of volumes of the (**a**) dorsal DG, CA3 and CA1; and of the (**d**) ventral DG, CA3 and CA1. (**b**) Stereological estimations of volumes from CA1 strata oriens, pyramidale and radiatum. (**e**) Outlining of the different subareas: DG, CA3 and CA1—diagrams were adapted from the Paxinos mouse brain atlas,^40^ corresponding brain slices were stained with Giemsa. Stereological estimations of cell numbers on the dorsal (**c**) and ventral (**f**) hippocampus (strata pyramidale for CA1 and CA3 and granule cell layer for DG). IFNγ KO *n*=5 and WT=5. Error bars denote s.e.m.; *P<0.05. DG, dentate gyrus; IFNγ, interferon-γ KO, knockout; WT, wild type.

As the only observed behavioural differences were in cognitive tasks that are known to be dependent on hippocampal functioning (particularly the dorsal region), we next measured volumes of different subregions of the dorsal (Figure 2a) and ventral (Figure 2b) hippocampus using a stereological approach. Our results show that IFNγ absence results in an increased volume of the dorsal CA1 region as compared with WT (*t*_(8)_=2.589; *P*=0.0322; *d*=1.64); of note, there was also a tendency for increased volumes in the dorsal DG of IFNγ KO mice (*t*_(8)_=1.695; *P*=0.1286.; *d*=1.0708), with a strong effect size, that did not, however, reach statistical significance (Figure 2a).

Within the dorsal CA1 region, IFNγ KO mice exhibited enlarged strata oriens (*t*_(8)_=2.763; *P*=0.02; *d*=1.75) and radiatum (*t*_(8)_=2.608; *P*=0.03; *d*=1.65) of CA1 with no statistically significant differences on stratum pyramidale (*t*_(8)_=1.891; *P*=0.0953; *d*=1.20) despite the strong effect size (Figures 2c and d). Importantly, in line with the results of the behavioural testing, there were no volumetric differences in any of the subregions of the ventral hippocampus (Figure 2b).

We also counted the number of neurons in the dorsal and ventral part of the hippocampus (Figures 2e and f) using an unbiased stereological approach and observed an increased number of cells in the dorsal CA1 stratum pyramidale (*t*_(8)_=2.700; *P*=0.0271; *d*=1.71) and a tendency for increased cell numbers in the dorsal DG granule cell layer (Figure 2e) from IFNγ KO mice (*t*(8)=1.973; *P*=0.0839; *d*=1.25). Curiously, no alterations were observed in the ventral region of the hippocampus (Figure 2f).

### Absence of IFNγ enhances neuroplastic phenomena in the dorsal hippocampus

**Figure 3:**
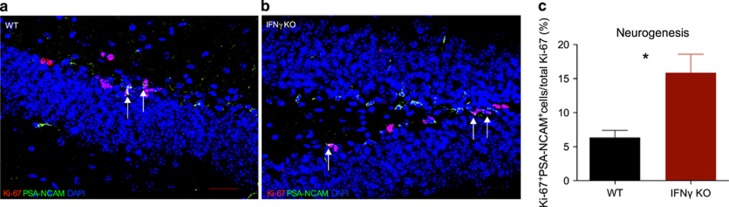
Neurogenesis is increased in the DG of the dorsal hippocampus of IFNγ KO mice. Photomicrograph depicts Ki-67 (red) and PSA-NCAM (green) double staining in the DG SGZ from WT (**a**) and IFNγ KO (**b**). Graph displays the percentage of newly born neurons (Ki-67^+^ cells that express PSA-NCAM^+^) by the total proliferating cells (Ki-67^+^) for assessing neurogenesis (differentiation) in the DG SGZ of the dorsal hippocampus (**c**). IFNγ KO *n*=3 and WT=3. Scale bar, 30 μm. Error bar denotes s.e.m. *P<0.05. DG, dentate gyrus; IFNγ, interferon-γ KO, knockout; PSA-NCAM, polysialic acid-NCAM antibody; SGZ, subgranular zone; WT, wild type.

As our stereological and behavioural results pointed to a specific effect on the dorsal hippocampus, we focused our subsequent analyses in this brain region. Neurogenesis, a process characterized by the generation of newly born neurons, is known to occur in the SGZ of the DG throughout life and is an important contributor for cognitive processes (reviewed by Kemmperman *et al.*^41^). As IFNγ is a known regulator of cell proliferation, we explored whether the effects observed above could also be related with altered hippocampal neurogenesis. To do so, we counted in the SGZ the percentage of proliferating cells (Ki-67^+^) also expressing the early neuronal marker polysialic acid-NCAM antibodies (double-labelled cells) in the SGZ. In accordance with our hypothesis, IFNγ KO mice displayed enhanced neurogenesis compared with WT (*t*_(4)_=3.239; *P*=0.03; *d*=2.64; Figures 3a–c).

**Figure 4:**
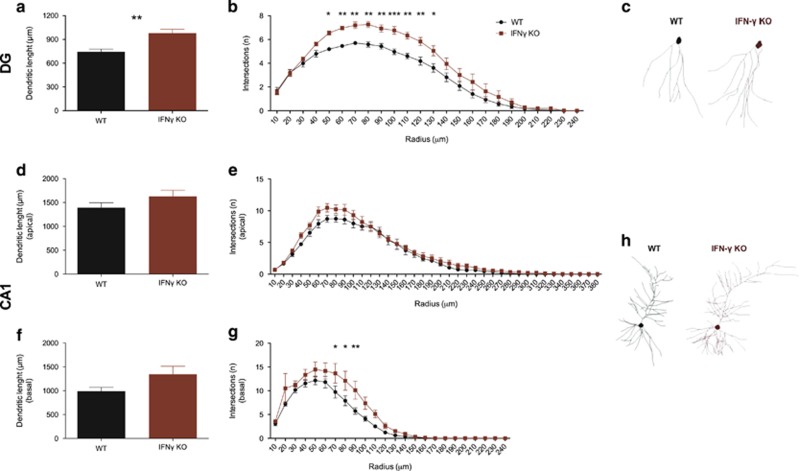
Neuronal dendritic trees are enlarged in IFNγ KO mice. Morphometric analysis of Golgi-stained hippocampal neurons of the: DG—total dendritic length (**a**), differential rearrangement of dendrites (**b**) and computer-assisted three-dimensional (3D) reconstructions of a representative granule neuron (**c**); CA1—total length of the apical (**d**) and basal dendrites (**f**), differential rearrangement of the apical (**e**) and basal (**g**) dendrites and computer-assisted 3D reconstructions of a representative pyramidal neuron (**h**). (IFNγ KO =5–6 and WT=6). Error bars denote s.e.m.; *P<0.05; **P<0.01 and ***P<0.001. DG, dentate gyrus; IFNγ, interferon-γ KO, knockout; WT, wild type.

Another neuroplastic phenomena known to modulate cognitive performance is dendritic remodelling. We observed that dendrites from dorsal DG granule neurons of mice that lack IFNγ are longer (total length: *t*_(10)_=3.888; *P*=0.003; *d*=2.24; Figure 3a) and more ramified than those of WT (Figures 4a–c), particularly at a distance between 50 and 130 μm from the soma (Sholl analysis: genotype—F_(1,10)_=11.98; *P*=0.0061; *η*^2^_p_=0.39, genotype × distance to soma—F_(23,230)_=3.817; *P*<0.0001; *η*^2^_p_=0.28). Although the total length of CA1 pyramidal neurons dendrites were not different between the two genotypes (Figure 4d, apical: *t*_(9)_=1.418; *P*=0.1899; *d*=0.85; Figure 4f, basal: *t*_(9)_=2.001; *P*=0.0764; *d*=1.17), there was a clear tendency, with a strong effect size, for basal dendrites of CA1 pyramidal neurons of IFNγ KO animals to be larger; in fact, they had significantly more branches, particularly at a distance between 70 and 90 μm near the soma (Sholl analysis: genotype—F_(1,9)=_3.553; *P*=0.0921; *η*^2^_p_=0.18, genotype × distance from soma—F_(23,207)_=2.406; *P*=0.0006; *η*^2^_p_=0.21) (Figures 4g and h).

**Figure 5:**
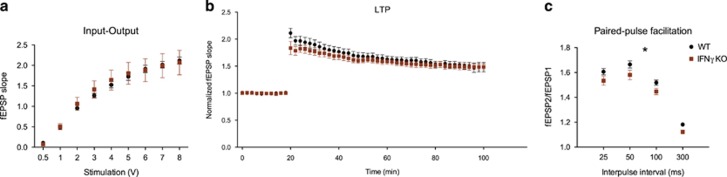
Pre-synaptic plasticity is altered in IFNγ KO mice. Electrophysiological analysis using dorsal hippocampal slices: input–output (**a**), long-term potentiation (LTP) response (**b**) and paired-pulse facilitation (**c**) assays. (IFNγ KO=8 and WT=9). Error bars denote s.e.m.; *P<0.05. IFNγ, interferon-γ KO, knockout; WT, wild type.

Together with structural plasticity, functional synaptic plasticity is the hallmark of learning and memory. To study the impact of the absence of IFNγ in functional synaptic plasticity, we recorded extracellular field potentials on fresh slices of the CA1 region of the dorsal hippocampus. To address both pre- and postsynaptic mechanisms, we analysed three plasticity-related electrophysiological phenomena: the input–output response (global), LTP induction after tetanus stimulation (mainly postsynaptic) and paired-pulse facilitation (mainly pre-synaptic). Although the first two phenomena were not different between IFNγ KO mice and WT (Figures 5a and b), the EPSP ratio in the paired-pulse facilitation paradigm was lower in IFNγ KO mice compared with WT (F_(1,15)_=5.104; *P*=0.039; *η*^2^_p_=0.48; Figure 5c), implying that IFNγ absence is associated with an increased pre-synaptic neurotransmitter release probability.

## Discussion

Herein, we have identified the pro-inflammatory cytokine, IFNγ, as a regulator of cognitive performance. Moreover, we have also identified that its effects are regionally selective as most alterations caused by its absence were allocated to the dorsal, but not the ventral, hippocampus. We also demonstrated the impact of the absence of this cytokine on neuroplastic phenomena that are known to support cognitive functioning, namely an increase in the generation of adult newly born neurons in the DG, enlarged dendritic arborizations and higher pre-synaptic vesicle release probability.

Of the several behavioural dimensions assessed, the absence of IFNγ selectively alters the performance on cognitive-related tasks and more specifically, on tasks engaging the hippocampus. Interestingly, the volumetric data, showing only alterations in the dorsal part of the hippocampus, support the cognitive specificity already noted in the behavioural results, as the dorsal hippocampus is functionally distinct of its ventral counterpart, assuming cognitive-related functions while the latter is responsible for emotional processing.^42^

The hippocampus has a central role in learning and the acquisition of new memories. Integrity of these functions requires ongoing neurogenesis in the (hippocampal) SGZ of the adult brain such that its pharmacological arrest results in long-term emotional and cognitive changes.^43, 44^ In accordance, here we demonstrate that IFNγ KO mice present higher numbers of adult newly born neurons in the DG. Of note, a previous study reported a decrease on doublecortin-positive cells in IFNγ KO mice.^45^ Although these results may apparently go in a different direction from ours, we cannot exclude that we are targeting a different subset of immature neurons, as only actively proliferating neuroblasts were considered. Moreover, the reduction of doublecortin-positive cells in IFNγ KO mice may reflect a reduced immature neuronal stage, with more neurons being integrated, although this needs further investigation.

In line with our results, data by Li *et al.*^9^ also showed an increase in neurogenesis in IFNγ KO mice with increased number of proliferating cells in the subventricular zone and an increased percentage of newly born neurons in the olfactory bulb.^9^ However, the novel finding that these mice also present more newly born neurons in the DG has a special relevance for cognitive function.

Of note, as IFNγ is a pro-inflammatory cytokine with an important function in the context of the immune response, it may be possible that IFNγ effect on neurogenesis in pathological contexts differs from that of the healthy brain. Indeed, in the paper by Baron *et al.*,^20^ it is shown that in an Alzheimer's disease model, genetically engineered for producing limited amounts of IFNγ, neurogenesis is enhanced. Here, the disease context, stage of pathogenesis and cellular/cytokine milieu may also dictate the disease resolving and/or contributory nature of IFNγ.

One of the key components of neuronal plasticity is the complexity of the dendritic arbour. Learning, for instance, shapes the dendritic morphology of recently generated neurons to integrate them into neuronal circuits.^46^ Previous studies have demonstrated that IFNγ by itself is capable of negatively shaped neuronal morphology.^6, 14^ In fact it was shown that exposing neurons in an *in vitro* culture to IFNγ inhibited dendritic outgrowth, induced dendritic tree retraction and diminished the rate of synapse formation.^6^ Adding to these previous observations, our current data reveal that the DG granule neurons from IFNγ KO mice present larger dendritic trees, which together with the increased proliferation of newly born neurons, contributes to the tendency for an increased volume observed in this hippocampal subregion. Likewise, the fact that the volume of strata oriens and radiatum from the CA1 region were also increased in IFNγ KO mice can be potentially attributed, at least in part, to the tendency for increased dendritic length of both the apical and basal dendritic tree in pyramidal neurons of this area. Apart from structural plasticity, functional plasticity at the synapse/circuit levels, as assessed by LTP and paired-pulse protocols, is also critical for learning and memory. We here show that the absence of IFNγ has no effect on LTP induction, as mice lacking this cytokine are equally capable of inducing an efficient hippocampal LTP response when compared with WT animals. Although this finding might seem in contradiction with the LTP impairment by acute IFNγ local administration,^7^ it might be because under basal conditions IFNγ has a very limited role in postsynaptic plasticity or simply reflect the development of adaptive mechanisms (as our model is a constitutional knockout). Yet, we have demonstrated that mice lacking IFNγ exhibit a decreased paired-pulse ratio without changes in the input/output curve. Importantly, although this is sometimes interpreted as decreased synaptic plasticity, it more probably reflects an increased pre-synaptic release probability.^47^ Of note, the co-existence of enhanced cognitive performance and decreased paired-pulse ratio observed in the present work is in line with preclinical models of Alzheimer's disease in which an increased paired-pulse ratio is one of the first signs of synaptic dysfunction and cognitive deficits.^48^

Given the fact that our mouse line is a constitutional deletion model, it is plausible that the structural, morphological and functional alterations herein observed may have been induced during neurodevelopmental stages. As an example, IFNγ has been implicated in the cognitive deficit of Down syndrome, a neurodevelopmental disorder characterized by an extra copy chromosome 21 (chromosome 16 in mice), which codifies for many IFN-regulated genes and IFN receptors,^49, 50^ with patients displaying increased levels of this cytokine^18^ and increased sensibility to IFN action.

Interestingly, although endogenous brain IFNγ is only very weakly expressed at basal conditions, here we show that its absence leads to important structural, morphological and functional alterations in the hippocampus that contribute to an enhanced cognitive performance. Therefore, our results suggest that in a pathological context, where IFNγ levels may be significantly altered, its role in mediating cognitive decline may emerge as particularly important to explore. Moreover, this cytokine and its downstream molecules may be promising therapeutic targets to prevent and/or treat cognitive decline associated with inflammation.
